# BondGraphs.jl: composable energy-based modelling in systems biology

**DOI:** 10.1093/bioinformatics/btad578

**Published:** 2023-09-19

**Authors:** Joshua Forrest, Vijay Rajagopal, Michael P H Stumpf, Michael Pan

**Affiliations:** School of BioSciences, University of Melbourne, Parkville, Australia; School of Mathematics and Statistics, University of Melbourne, Parkville, Australia; Department of Biomedical Engineering, University of Melbourne, Parkville, Australia; Department of Biomedical Engineering, University of Melbourne, Parkville, Australia; Baker Department of Cardiometabolic Health, University of Melbourne, Parkville, Australia; The Graeme Clark Institute, University of Melbourne, Parkville, Australia; School of BioSciences, University of Melbourne, Parkville, Australia; School of Mathematics and Statistics, University of Melbourne, Parkville, Australia; Melbourne Integrative Genomics, University of Melbourne, Parkville, Australia; ARC Centre of Excellence for the Mathematical Analysis of Cellular Systems, University of Melbourne, Parkville, Australia; School of Mathematics and Statistics, University of Melbourne, Parkville, Australia

## Abstract

**Summary:**

BondGraphs.jl is a Julia implementation of bond graphs. Bond graphs provide a modelling framework that describes energy flow through a physical system and by construction enforce thermodynamic constraints. The framework is widely used in engineering and has recently been shown to be a powerful approach for modelling biology. Models are mutable, hierarchical, multiscale, and multiphysics, and BondGraphs.jl is compatible with the Julia modelling ecosystem.

**Availability and implementation:**

BondGraphs.jl is freely available under the MIT license. Source code and documentation can be found at https://github.com/jedforrest/BondGraphs.jl.

## 1 Introduction

The last decade has seen an explosion of experimental methods that simultaneously measure many aspects of biological processes at multiple temporal and spatial scales. Extracting biological mechanisms from this big-data explosion requires modular, interoperable, and computationally efficient software in systems biology (Rajagopal *et al.* 2022).

Bond graph modelling is a promising approach to creating computational models for large-scale biological data analysis. This mathematical framework has been used to model many biological systems, as well as mechanical, electrical, and chemical engineering systems (Gawthrop and Bevan 2007, Gawthrop and Pan 2022).

Bond graph models are particularly advantageous for systems biologists and mathematical modellers. Bond graph models of biological networks are biophysics-based and are scalable to large networks of interactions. They satisfy the laws of thermodynamics implicitly, and therefore they enable efficient construction of models that couple interactions between different subsystems within a biological process. Recent biological bond graph models include mitochondrial respiration (Gawthrop *et al.* 2020), two component bacterial systems (Forrest *et al.* 2023), and vascular blood flow (Safaei *et al.* 2018).

Bond graphs are most useful when the system being modelled satisfies one or more of the following: (i) different types of physics are involved (such as an electrochemical system), (ii) physical consistency is required on a large scale, (iii) the system exhibits a modular or hierarchical structure, and (iv) model compartments are usable in other models. Thus, bond graphs are a promising approach to improving model reusability and model composition in systems biology.

Here, we present **BondGraphs.jl**, a Julia implementation of the bond graph modelling framework. The package enables a wide range of analyses to be conducted on physical systems, including the derivation of differential equations and simulation. Graphs and equations are built with a high-level symbolic interface that integrates closely with the Julia modelling ecosystem.

## 2 Features

The most pertinent features are listed below. We refer to the documentation (https://jedforrest.github.io/BondGraphs.jl/stable/) for a full list of features and examples and to the Supplementary Material for the basic principles of bond graph modelling.

### 2.1 Graphical construction and modification of models

One of the strengths of bond graphs is that they are semantic representations of models, much like an electrical circuit, free body diagram, or reaction network. Physical systems are represented as graphs and can be manipulated in many of the same ways.

BondGraphs.jl constructs a **BondGraph** object using **Component**s and **Junction**s (graph vertices) and **Bond**s (graph edges).

Each **Component** encodes a set of acausal constitutive equations that represent a physical element, event, or law; e.g. Ohm’s law, or mass action kinetics. **Junction**s are vertices that describe how components relate to each other according to conservation laws; e.g. Kirchhoff’s Voltage Law or mass balance in biochemistry. **Bond**s are the graph edges that connect the vertices and represent energy flow or transfer between parts of the system.

Bond graphs can either be constructed one component at a time and connected together with bonds (using **add_node!** and **connect!**) or generated automatically using specialized algorithms (see Section 2.3).

Extra functions are included for modifying and composing multiple bond graph objects. Vertices (components or junctions) can be swapped in for another (**swap!**). This is useful for rapidly creating bond graphs of systems that are structurally similar but with modified equations, such as a different constitutive relation for a spring or resistor. For example, in a biochemical context, one could replace standard mass-action rate laws with Michaelis–Menten rate laws. In fact, any arbitrary rate law can be used, so long as it is thermodynamically consistent (Pan *et al.* 2021).

Vertices can also be inserted between two already connected components (**insert_node!**), which is useful for adding a conservation law (with a junction) or a transformation to the variables.

When combining two separate bond graphs, there are two main approaches. The first is to take the two disconnected graphs and merge nodes representing the same object (**merge_nodes!**). This way the bond graph retains a ‘flat’ structure and system elements are not duplicated. Alternatively, a bond graph can be nested inside a **BondGraphNode** component, which functions like an ordinary component but contains another bond graph. This creates a hierarchical bond graph that is a powerful tool for model organization (Pan *et al.* 2021).

These operations enable high-level semantic construction and modification of the model. Since all models are built and stored as a graph object, they can be traversed and manipulated using existing graph algorithms (see Section 2.4). The bond graph plots in this article, for example, use a graph layout plotting algorithm.

BondGraphs.jl includes a library of common bond graph component types. The default library includes a biochemical library for modelling chemical species and reaction networks. Additional custom components can be defined by the user and stored in the local library (see Supplementary Material for further details).

### 2.2 Symbolic generation of equations

Systems of differential equations are automatically generated from the bond graph. Stored equations are systematically composed using a computer algebra system (CAS) to automatically substitute, simplify, and differentiate equations. This produces a system of ordinary differential equations (ODEs) or differential algebraic equations. These equations can be solved numerically or analysed analytically using other packages in the Julia ecosystem (Section 2.4). A modeller is not limited to differential equations, as they can derive other useful representations such as Hamiltonians, reaction fluxes, or power equations.

A user is free to specify parameter values at multiple stages during the modelling process. Parameter values can be set during initialization using the constructor syntax: **Component(**:C, “membrane”; C = 1) or modified later using Julia’s indexing syntax: **membrane****.**C = 1.

The benefit of this approach is that the model building and equation solving steps are separated. Abstractions of models and repeated motifs can be stored and reused for different modelling contexts (Gawthrop and Bevan 2007, Pan *et al.* 2021). The parameter values can be decided at the simulation stage, and the CAS simplification routines reduce numerical instability. With Julia, arbitrary functions can be used as control inputs, as long as they return a single real-valued output.

### 2.3 Generating models from chemical reaction networks

An important feature for systems biologists is the chemical reaction network → bond graph → differential equation pipeline. BondGraphs.jl provides an interface for Catalyst.jl (Loman *et al.* 2022) which converts a reaction network into a bond graph. This enables rapid construction and analysis of models without needing to manipulate the graph structure or type equations directly (though this is still possible if desired) (see Section 3.2 for an example). Existing models that were originally represented as chemical reaction networks can therefore be easily converted into bond graphs.

### 2.4 Integration with the Julia modelling ecosystem

Previous bond graph packages have largely been implemented through proprietary software languages such as MATLAB or Maple. More recently, the open source package BondGraphTools implemented the framework in Python (Cudmore *et al.* 2021). However, since bond graphs are implemented as specialized Python objects, it is not possible to use or study the graph-theoretic properties of the model, or to easily integrate with other modelling or analysis libraries.

**Figure 1.**
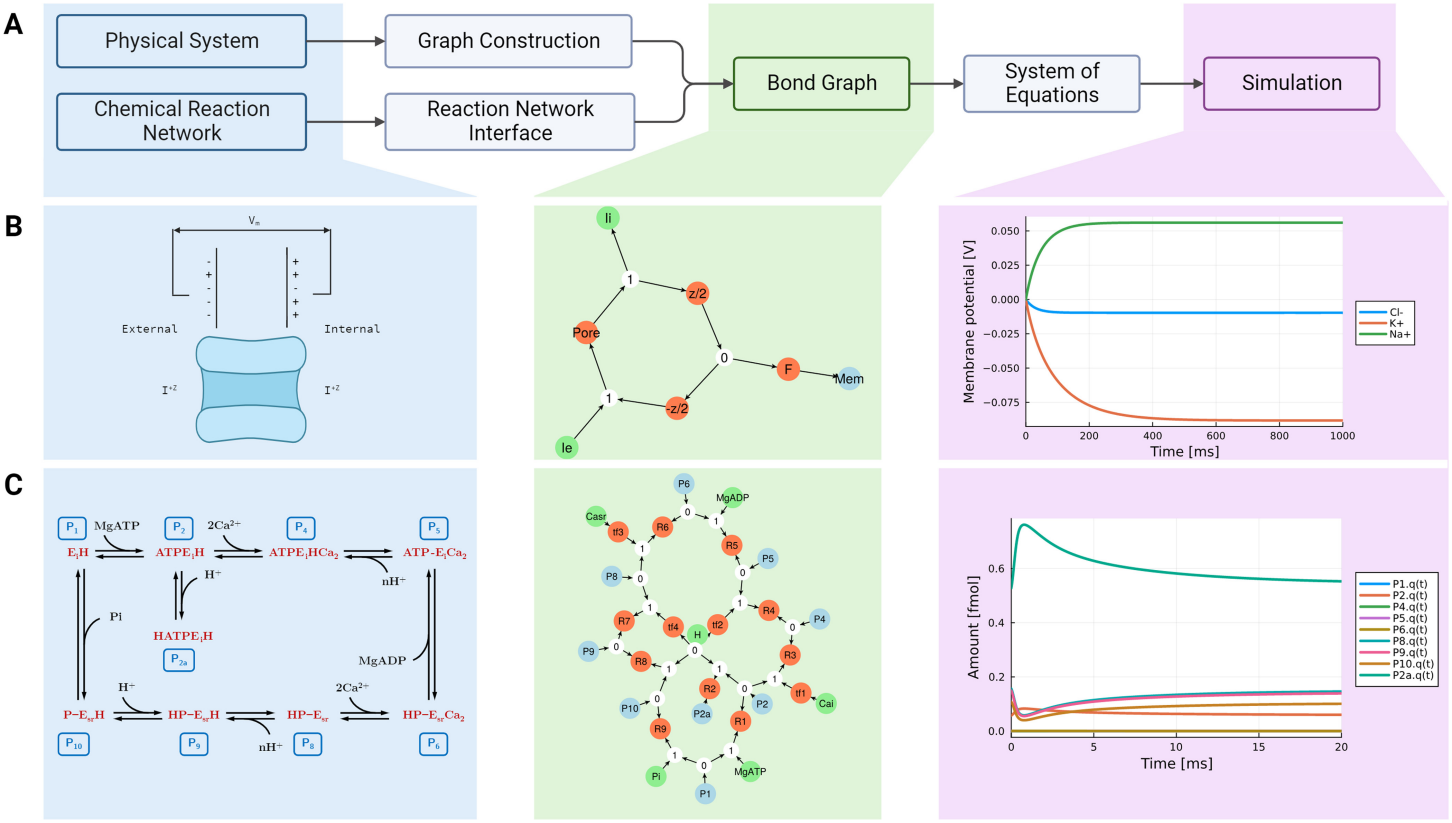
The BondGraphs.jl modelling process. (A) A physical system can be represented as a bond graph using either component-wise construction or, if it is a chemical reaction network, using the reaction network interface. BondGraphs.jl includes a plotting recipe for bond graphs: chemical species (blue), chemostats (green), reactions **Re** and transformers **TF** (orange), and junctions (white). The most common use case is to use the bond graphs to generate a symbolic system of equations that can be solved numerically. (B) The ion pore channel (Cudmore *et al.* 2021) demonstrates the modelling of a multiphysics (electrochemical) system. (C) The SERCA pump (Pan *et al.* 2019) demonstrates the use of the reaction network interface and scalability of Bond Graphs. Code used to generate the bond graphs and numerical solutions is available in the Supplementary Material. Diagrams created with BioRender.com.

In contrast, BondGraphs.jl is built purely in Julia. Julia is computationally fast and scalable while retaining easy-to-read syntax. It is therefore, we would argue, better positioned to address the grand challenges of systems biology (Roesch *et al.* 2023). The data types and functionality of BondGraphs.jl are built upon widely used packages, which give users access to all their features. This also enables close integration with the Julia modelling ecosystem. **BondGraph**s are **AbstractGraph**s as defined in Graphs.jl (https://github.com/JuliaGraphs/Graphs.jl/). With this, we can use a library of graph operations, traversal algorithms, graph colouring, database storage, and plotting recipes (as seen in Fig. 1). ODEs are generated and stored using ModelingToolkit.jl (Ma *et al.* 2022) and are solved with the parallelizable numerical solver library DifferentialEquations.jl (Rackauckas and Nie 2017). Bridging BondGraphs and ModelingToolkit connects our bond graphs to many other useful libraries, including parameter estimation and optimization, plotting libraries, reaction network interfaces (Section 2.3), and standard model formatting and parsing (e.g. SBMLToolkit, part of ModelingToolkit, see Supplementary Material). This also makes the package code easier to maintain, and updates to other packages will automatically be available for use in BondGraphs.jl.

## 3 Results

Here, we demonstrate the features of BondGraphs.jl through two biological examples from literature. These two examples highlight several important features of both bond graphs and BondGraphs.jl. A further large-scale model (281 species, 566 reactions) of a yeast metabolic network is included in the Supplementary Material. All code used to generate these plots and results are found in the Supplementary Material.

### 3.1 Ion transport

Many biological systems in nature involve multiple processes described by different physics. Here, we model an ion pore transport process, which couples electrical and chemical processes that drive the movement of ions across cell membranes (Cudmore *et al.* 2021). The diagram, bond graph, and numerical solution are shown in Fig. 1A.

Each vertex in the bond graph (middle column) represents a different physical element or law of the system. The membrane potential is an electrical capacitor (blue). The external and internal ion concentrations are constant sources of chemical energy (green). Orange vertices are two-port components that either ‘transform’ or ‘dissipate’ energy: the energy-dissipating ion transport reaction process (*pore*); the conversion of electrical to chemical potential (*F*); and the splitting of voltage and charge dependence across the two sides of the membrane (*z/2* and −*z/2*). The junctions (0 and 1) are conservation laws: 0-junctions represent equal effort (typically 0D) and 1-junctions represent equal flow (1D or greater). In these examples, they are the conservation of molar flow rate (0) and chemical potential (1). Bonds (edges) are the flow of energy between components, with the arrowhead indicating sign convention.

Components are constructed with parameter values and initial conditions; however, these can be determined later. All nodes are added to the bond graph and connected with bonds in a graph-like manner. Once the bond graph is made, it can easily be reused with different values for different ions. Solutions for three ion transporters (Na${}^{+}$, K${}^{+}$, and Cl${}^{-}$) are included in Fig. 1A, right column. The steady-state solutions follow the well-known Nernst equations seen in electrophysiology (Cudmore *et al.* 2021).

### 3.2 Sarco/endoplasmic reticulum Ca^2+^-ATPase

Figure 1 shows a bond graph construction of the sarco/endoplasmic reticulum Ca^2+^-ATPase (SERCA), first presented by Tran *et al.* (2009) and subsequently represented as a bond graph in Pan *et al.* (2019). Reaction network and parameter values are taken from these papers. Figure 1B shows the reaction network schematic, bond graph, and numerical solution for species’ concentrations over time.

The colour coding follows the same convention as the previous example: blue vertices store chemical energy for one species; green vertices are sources of chemical energy (chemostats); orange vertices are reactions; junctions are physical conservation laws.

The SERCA bond graph model is generated using the reaction network interface (Section 2.3) with the following compact syntax:


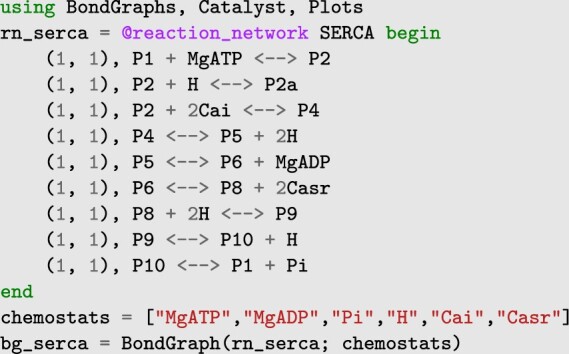


Note that the **(1, 1)** before each equation are placeholder reaction rates required for the catalyst interface.

This example highlights several features: (i) model construction scales efficiently even for large complex reaction networks, (ii) bond graphs can be constructed from only the reactions network and interfaced at a high level without direct interaction with equations, and (iii) parameter values and initial conditions are determined separately and can include control variable equations; the input concentration of Ca^2+^ is set as a nonlinear ‘spike’ function over time (see Supplementary Material for details).

## 4 Conclusion

BondGraphs.jl is a new powerful tool for large, multiphysics, biophysically constrained systems biology models. Bond graphs are a general framework beyond biology; however, they have specific benefits to the systems biologist: (i) models are constructed abstractly without worrying about equation implementation, (ii) governing equations are derived automatically, (iii) the energy-based framework ensures that generated models are physically and thermodynamically compliant, and (iv) BondGraphs.jl naturally integrates with other state-of-the-art tools in the Julia modelling ecosystem.

## Supplementary Material

btad578_Supplementary_Data

## Data Availability

The code used to generate the results are available in the supplementary material and online (https://jedforrest.github.io/BondGraphs.jl/stable/).
