# Supplementary material for: BondGraphs.jl: composable energy-based modelling in systems biology
Source: Bioinformatics. 2023 Sep 19;39(10):btad578. doi: 10.1093/bioinformatics/btad578 (PMC10551222; doi:10.1093/bioinformatics/btad578)
Supplement: btad578_Supplementary_Data [file btad578_supplementary_data.pdf]

Supplementary material for "BondGraphs.jl: Composable energy-based modelling in systems biology"

A. Bond graph theory

This section covers the basic principles of bond graph modelling. Table 1 summarises the variable and component analogies discussed in this section.

Construction

Bond graphs represent all systems and reactions in terms of energy flow between elements of a system. Physical variables are described in terms of abstracted *efforts* (forces, voltages, chemical potentials) and *flows* (velocities, currents, molar flow rate). Efforts are always in terms of Joules per some quantity x, and flows are always x per second, so that $J/x \cdot x/s = J/s$, or energy change over time. By abstracting these units, any of the above efforts and flows can be combined in a single model that conserves energy across all domains.

Bond graphs are built with collections of components. Components are generalised forms of physical laws and typically represent a physical element of the system, such as a resistor, point mass, or chemical species. Each component stores a constitutive relation that describes the relationship between the energetic *effort* [Joules/x] and energy *flow* [x/second] in the component, where x is a measured quantity (displacement, charge, moles). BondGraphs.jl includes a library of common bond graph component types. These include the **C** type components (capacitors, chemical species), **R** type components (resistors, reactions), **I** type components (inductors, point masses), and energy sources (batteries, chemostats). For more examples and details of bond graph components, refer to Gawthrop and Bevan (2007) and Borutzky (2011).

The biochemical reaction component **Re** differs from the standard **R** component in that it is a non-linear function of two efforts (chemical potentials). In other words, a distinction is made between the potential of the *reactants* and the potential of the *products*. In bond graph terms, the **Re** component has two ports instead of one. In other aspects the analogy with the resistive **R** component still holds, as they are all energy 'sinks' through the release of thermal energy.

The standard constitutive equations for a biochemical bond graph produce mass-action rate laws. Other rate laws may be specified. In the BondGraphs.jl documentation there is an example of implementing a Michaelis-Menten rate law in place of mass-action kinetics (`https://jedforrest.github.io/BondGraphs.jl/stable/examples/`). Any rate law can be used in principle, so long as it is thermodynamically consistent.

Components are joined with a bond, representing the energy transfer between components. Each bond has an associated effort and flow variable and a direction that determines sign convention. Bonds may also connect to junctions, which describe particular conservation laws. These are either *Equal Effort* (effort is equal in connected components) or *Equal Flow* (flow is identical). In bond graph notation, these are equivalent to the **0** and **1** junctions respectively. Intuitively, these can be thought of as junctions that conserve the (typically) 0D effort and 1D flow.

Table 1. Bond graph variables and analogous components for common physical domains.

	Bond Graph	Mechanical	Electrical	Chemical
Quantity [x]	x	[metres]	[coulombs]	[moles]
Effort [Joules/x]	e	Force	Voltage	Chemical potential
Flow [x/second]	f	Velocity	Current	Molar flow rate
Integrated Effort	$p = \int edt$	Momentum	Magnetic flux	*n/a*
Integrated Flow	$q = \int fdt$	Displacement	Charge	Concentration
Compliance	**C**	Spring	Capacitor	Species
Resistance	**R**	Damper	Resistor	Reaction
Inductance	**I**	Inertia	Inductor	*n/a*
Transform	**TF**	Lever	Transformer	Stoichiometry

Thermodynamic consistency

Since bonds are two-way energy connectors, the energy of the entire system is always conserved. (Assuming a closed system. In an open system, energy can be dissipated or added, but it can always be tracked.) A similar rule applies to mass conservation or charge conservation. Bond graphs therefore by design enforce physical and thermodynamic constraints. More precisely, bond graphs satisfy energy conservation as all bonds and junctions are power conserving. While entropy is not always explicitly shown, the components can be modified to account for temperature and entropy flow (for example in Borutzky, 2011).

However, it is up to the modeller to ensure that the components are 'well formed' and physically plausible. In other words, if an arbitrary component's constitutive law does not conserve energy (for example, through the use of irreversible rate laws), then the system as a whole will not conserve energy. Nonetheless, the bonds, ports, and junctions in the bond graph framework are thermodynamically safe. Thus, model composition across scales and domains will not break physical constraints. This makes it possible to construct realistic large-scale biophysical models.

Therefore, there is some onus on the modeller to ensure that newly designed components are physically plausible. However, existing standard components (such as the Reaction component **Re**) are physically plausible. Pre-made components in the BondGraphs.jl library all satisfy the laws of thermodynamics.

Specific to biochemistry, bond graphs are able to automatically satisfy detailed balance constraints in chemical reaction network models. This is in contrast to typical mass action models with kinetic parameters, where additional detailed balance constraints need to be identified and enforced (Liebermeister and Klipp, 2006). Bond graphs assign a parameter for each species (thermodynamic constant $K_i[\mathrm{mol}^{-1}]$) and reaction (reaction rate $\kappa_j[\mathrm{mol} \cdot \mathrm{s}^{-1}]$) in the system. In contrast, kinetic parameters in conventional models are combinations of thermodynamic parameters, and changing the value of one kinetic parameter without accounting for changes in the other kinetic rates may result in a thermodynamically infeasible system. Previous bond graph papers have addressed this issue (Pan et al., 2019; Gawthrop et al., 2021) and Mason and Covert (2019) benchmark the fitting of thermodynamic parameters to data compared to conventional kinetic parameters.

Bond graphs have the additional benefit of reducing the parameter space to thermodynamically feasible regions. In this sense, they have a similar benefit to convenience kinetics and similar frameworks. A discussion about convenience kinetics and bond graphs is given in Pan et al. (2021).

For further reading on how bond graphs satisfy detailed balance, see Pan et al. (2019). For a discussion on dealing with uncertainty in thermodynamic parameters, we refer to Gawthrop et al. (2015).

B. Model descriptions

Model specifications and code for the two models used in Figure 1 in the Section 3 of the main text: the ion pore transport model (Cudmore et al., 2021), and; the Sarco/Endoplasmic Reticulum $Ca_2{}^{+}$-ATPase (SERCA) pump (Pan et al., 2019).

We have included an additional example on a large (281 species, 566 reactions) *Saccharomyces cerevisiae* (yeast) metabolic network (Stanford et al., 2013). This example features automatically generating a bond graph from a published systems biology model stored in BioModels (Malik-Sheriff et al., 2020).

Each section includes the generated ODE system of equations (or a subset of the equations), model parameters and initial conditions (where applicable), and the Julia code used to create the bond graph models and plots in Figure 1.

B.1. Ion Transport

B.1.1. ODE

$$\frac{\mathrm{d}V_m}{\mathrm{d}t} = \frac{zFr}{C_m}\left[K_e c_e \exp\left(\frac{-zFV_m}{2RT}\right) - K_i c_i \exp\left(\frac{zFV_m}{2RT}\right)\right]$$

V_m is the membrane voltage, z is the ion charge (here ± 1), $C_m = 1\ \mu\mathrm{F/cm}^2$ is the membrane capacitance, $F = 96485$ C/mol is the Faraday constant, $R = 8.314$ J/mol/K is the molar gas constant, T is the absolute temperature, K_i, K_e are the internal/external thermodynamic constants, and c_i, c_e are the internal/external ion concentrations.

B.1.2. Parameters and Initial Conditions

Table 2. Internal and external concentrations and charges for the ions Na^+, K^+, and Cl^-. Taken from Cudmore et al. (2021).

Ion	charge z	c_{in} [M]	c_{ex} [M]
Na^+	$+1$	0.019	0.155
K^+	$+1$	0.136	0.005
Cl^-	-1	0.078	0.112

B.1.3. Julia Code

```
using BondGraphs, Plots, GraphMakie
import CairoMakie as CM

# Define a function to create a bond graph for each ion
function ion_pore(name=""; z=1, c_ex=1e-3, c_in=1e-3)
    bg = BondGraph(name * " Ion Transport")

    # Create components
    membrane = Component(:C, "Mem"; C=1)
    ion_ex = Component(:SCe, "Ie"; K=1, xs=t->c_ex)
    ion_in = Component(:SCe, "Ii"; K=1, xs=t->c_in)
    potential_mem = EqualEffort()
```

```
    flow_f, flow_r = EqualFlow(), EqualFlow()
    TF_F = Component(:TF, "F"; n=96485)
    TF_zf = Component(:TF, "-z/2"; n=-z/2)
    TF_zr = Component(:TF, "z/2"; n=z/2)
    re_pore = Component(:Re, "Pore"; r=1e-7)

    # Add and connect components within bond graph
    allcomps = [
        membrane, ion_ex, ion_in,
        potential_mem, flow_f, flow_r,
        TF_F, TF_zr, TF_zf, re_pore
    ]
    add_node!(bg, allcomps)
    connect!(bg, ion_ex, flow_f)
    connect!(bg, flow_f, re_pore)
    connect!(bg, re_pore, flow_r)
    connect!(bg, flow_r, ion_in)
    connect!(bg, flow_r, (TF_zr,2))
    connect!(bg, (TF_zr,1), potential_mem)
    connect!(bg, potential_mem, (TF_zf,1))
    connect!(bg, (TF_zf,2), flow_f)
    connect!(bg, potential_mem, (TF_F,2))
    connect!(bg, (TF_F,1), membrane)
    return bg
end

# Plot an ion pore bond graph
ion_pore_bg = ion_pore()
fig = graphplot(ion_pore_bg)
CM.save("ion_transport_bg.svg", fig)

# Ion specific parameters
ions = Dict(
    "Na+" => (z=1, c_ex=155/1000, c_in=19/1000),
    "Cl-" => (z=-1, c_ex=112/1000, c_in=78/1000),
    "K+"  => (z=1, c_ex=5/1000, c_in=136/1000),
)

# Solve and plot each solution for the membrane potential
plot()
for (ionname, params) in ions
    model = ion_pore(ionname; params...)
    sol = simulate(model, (0., 1000.))
    plot!(sol, label=ionname, lw=3, xlabel="Time [ms]", ylabel="Membrane potential [V]")
end
plot!(; legend=:topright)
```

B.2. SERCA

B.2.1. Chemical Equations

$$\mathrm{P_1 + MgATP} \xrightleftharpoons{\mathrm{r_1}} \mathrm{P_2}$$

$$\mathrm{P_2 + H} \xrightleftharpoons{\mathrm{r_2}} \mathrm{P}_{2a}$$

$$\mathrm{P_2} + 2\,\mathrm{Ca}_i \xrightleftharpoons{\mathrm{r_3}} \mathrm{P_4}$$

$$\mathrm{P_4} \xrightleftharpoons{\mathrm{r_4}} \mathrm{P_5} + 2\,\mathrm{H}$$

$$\mathrm{P_5} \xrightleftharpoons{\mathrm{r_5}} \mathrm{P_6 + MgADP}$$

$$\mathrm{P_6} \xrightleftharpoons{\mathrm{r_6}} \mathrm{P_8} + 2\,\mathrm{Ca_{sr}}$$

$$\mathrm{P_8} + 2\,\mathrm{H} \xrightleftharpoons{\mathrm{r_7}} \mathrm{P_9}$$

$$\mathrm{P_9} \xrightleftharpoons{\mathrm{r_8}} \mathrm{P_{10} + H}$$

$$\mathrm{P_{10}} \xrightleftharpoons{\mathrm{r_9}} \mathrm{P_1 + Pi}$$

B.2.2. ODE

$$\begin{aligned}
\frac{\mathrm{d[P_1]}}{\mathrm{d}t} =& r_9\left(K_{10}[\mathrm{P_{10}}] - K_1K_{Pi}[\mathrm{P_1}][\mathrm{Pi}]_0\right) - r_1\left(K_{\mathrm{MgATP}}K_1[\mathrm{MgATP}]_0[\mathrm{P_1}] - K_2[\mathrm{P_2}]\right)\\
\frac{\mathrm{d[P_2]}}{\mathrm{d}t} =& r_1\left(K_{\mathrm{MgATP}}K_1[\mathrm{MgATP}]_0[\mathrm{P_1}] - K_2[\mathrm{P_2}]\right) - r_2\left(-K_{P2a}[\mathrm{P_{2a}}] + K_{\mathrm{H}}K_2[\mathrm{H}]_0[\mathrm{P_2}]\right) - r_3\left(-K_4[\mathrm{P_4}] + K^2_{\mathrm{Ca_i}}K_2[\mathrm{Ca_i}]^2_0[\mathrm{P_2}]\right)\\
\frac{\mathrm{d[P_{2a}]}}{\mathrm{d}t} =& r_2\left(-K_{P2a}[\mathrm{P_{2a}}] + K_{\mathrm{H}}K_2[\mathrm{H}]_0[\mathrm{P_2}]\right)\\
\frac{\mathrm{d[P_4]}}{\mathrm{d}t} =& r_3\left(-K_4[\mathrm{P_4}] + K^2_{\mathrm{Ca_i}}K_2[\mathrm{Ca_i}]^2_0[\mathrm{P_2}]\right) + r_4\left(-K_4[\mathrm{P_4}] + K^2_{\mathrm{H}}K_5[\mathrm{H}]^2_0[\mathrm{P_5}]\right)\\
\frac{\mathrm{d[P_5]}}{\mathrm{d}t} =& r_5\left(-K_5[\mathrm{P_5}] + K_{\mathrm{MgADP}}K_6[\mathrm{MgADP}]_0[\mathrm{P_6}]\right) + r_4\left(K_4[\mathrm{P_4}] - K^2_{\mathrm{H}}K_5[\mathrm{H}]^2_0[\mathrm{P_5}]\right)\\
\frac{\mathrm{d[P_6]}}{\mathrm{d}t} =& r_5\left(K_5[\mathrm{P_5}] - K_{\mathrm{MgADP}}K_6[\mathrm{MgADP}]_0[\mathrm{P_6}]\right) - r_6\left(K_6[\mathrm{P_6}] - K^2_{\mathrm{Ca_{sr}}}K_8[\mathrm{Ca_{sr}}]^2_0[\mathrm{P_8}]\right)\\
\frac{\mathrm{d[P_8]}}{\mathrm{d}t} =& r_6\left(K_6[\mathrm{P_6}] - K^2_{\mathrm{Ca_{sr}}}K_8[\mathrm{Ca_{sr}}]^2_0[\mathrm{P_8}]\right) - r_7\left(-K_9[\mathrm{P_9}] + K^2_{\mathrm{H}}K_8[\mathrm{H}]^2_0[\mathrm{P_8}]\right)\\
\frac{\mathrm{d[P_9]}}{\mathrm{d}t} =& r_7\left(-K_9[\mathrm{P_9}] + K^2_{\mathrm{H}}K_8[\mathrm{H}]^2_0[\mathrm{P_8}]\right) - r_8\left(K_9[\mathrm{P_9}] - K_{\mathrm{H}}K_{10}[\mathrm{H}]_0[\mathrm{P_{10}}]\right)\\
\frac{\mathrm{d[P_{10}]}}{\mathrm{d}t} =& r_8\left(K_9[\mathrm{P_9}] - K_{\mathrm{H}}K_{10}[\mathrm{P_{10}}]\right) - r_9\left(K_{10}[\mathrm{P_{10}}] - K_1K_{Pi}[\mathrm{P_1}][\mathrm{Pi}]_0\right)
\end{aligned}$$

Concentrations P_i and reaction rates r_j correspond to the chemical reaction network above. K_i is the thermodynamic constant for species i. Chemostats X are chemical species where the concentration is assumed constant ($\dot{X} = 0$). $[X]_0$ are the chemostat concentrations.

B.2.3. Parameters and Initial Conditions

Table 3. Parameter values and initial conditions for the states used in the SERCA bond graph model. Taken from Pan et al. (2019). The input function for Casr was chosen to resemble a physiological calcium spike, characterised by an initial spike in the concentration of Ca^{2+} followed by an exponential decay.

Reaction	Rate [fmol·s^{-1}]
r_1	0.00053004
r_2	8326784.0537
r_3	1567.7476
r_4	1567.7476
r_5	3063.4006
r_6	130852.3839
r_7	11612934.8748
r_8	11612934.8748
r_9	0.049926

Species	Affinity [fmol^{-1}]
P1	5263.6085
P2	3803.6518
P2a	3110.4445
P4	16520516.1239
P5	0.82914
P6	993148.433
P8	37.7379
P9	2230.2717
P10	410.6048
Cai	1.9058
Casr	31.764
MgATP	244.3021
MgADP	5.8126e-7
Pi	0.014921
H	1862.5406

Species	$x(t = 0)$ [fmol]
P1	0.000483061870385487
P2	0.0574915174273067
P2a	0.527445119834607
P4	1.51818391164022e-09
P5	0.000521923287622898
P6	7.80721128535043e-05
P8	0.156693953834181
P9	0.149232225342376
P10	0.108044124948978

Chemostat	Concentration [fmol]
Cai	0.0057
H	0.004028
MgADP	1.3794
MgATP	3.8
Pi	570
Casr	$\frac{2.5\sin(0.2t)}{(t^2+0.5)} + 0.2\exp\left(\frac{-t}{100}\right)$

B.2.4. Julia Code

```julia
using BondGraphs, Catalyst, Plots, GraphMakie
import CairoMakie as CM
import DifferentialEquations: Rosenbrock23

# Generate the bond graph from a chemical reaction network
rn_serca = @reaction_network SERCA begin
    (1, 1), P1 + MgATP <--> P2
    (1, 1), P2 + H <--> P2a
    (1, 1), P2 + 2Cai <--> P4
    (1, 1), P4 <--> P5 + 2H
    (1, 1), P5 <--> P6 + MgADP
    (1, 1), P6 <--> P8 + 2Casr
    (1, 1), P8 + 2H <--> P9
    (1, 1), P9 <--> P10 + H
    (1, 1), P10 <--> P1 + Pi
end
chemostats = ["MgATP", "MgADP", "Pi", "H", "Cai", "Casr"]
bg_serca = BondGraph(rn_serca; chemostats)
fig = graphplot(bg_serca)
CM.save("serca_bg.svg", fig)

# Define reaction rates, affinities, chemostats, and initial conditions
ca_spike(t) = 2.5*sin(t/5)/(t^2 + 0.5) + 0.2*exp(-t/100)
reaction_rates = [
    :R1 => 0.00053004,
    ... # defined as in above table
]
```

```
species_affinities = [
    :P1 => 5263.6085,
    ... # defined as in above table
]
chemostat_amounts = [
    :Casr => t -> ca_spike(t),
    ... # defined as in above table
]
initial_conditions = [
    :P1 => 0.000483061870385487,
    ... # defined as in above table
]

for (reaction, rate) in reaction_rates
    getproperty(bg_serca, reaction).r = rate
end
for (species, affinity) in species_affinities
    getproperty(bg_serca, species).K = affinity
end
for (chemostat, amount) in chemostat_amounts
    getproperty(bg_serca, chemostat).xs = amount
end
for (species, ic) in initial_conditions
    getproperty(bg_serca, species).q = ic
end

# Solve the differential equations generated by the bond graph
sol = simulate(bg_serca, (0., 20.); solver=Rosenbrock23())
plot(sol, lw=3, xlabel="Time [ms]", ylabel="Amount [fmol]", legend=:right)
savefig("serca_numerical_solution.svg")
```

B.3. *Saccharomyces cerevisiae* Metabolic Network

This example demonstrates how one could generate a bond graph model of a large biological network stored in a computer-readable format. For this example, we have used the *Saccharomyces cerevisiae* metabolic network from Stanford et al. (2013). This model contains 281 species and 566 reactions. The SBML model file contains information on relevant chemical species, reaction stoichiometry, default parameter values, and various other metadata. The file is downloadable from `https://doi.org/10.1371/journal.pone.0079195.s002`.

The bond graph is constructed using the chemical reaction network interface in BondGraphs.jl. The bond graph in total contains 1019 components, 1127 junctions, and 3813 bonds. We assume mass-action rate laws for simplicity and demonstration purposes; in practice one may update the **Re** reaction components with more sophisticated rate laws.

B.3.1. ODE

The system of ODEs generated by BondGraphs.jl has 293 equations. For the sake of conciseness, only one representative equation is shown here.

$$
\begin{aligned}
&\vdots \\
\frac{\mathrm{d}S_{0616}}{\mathrm{d}t} &= r_{69}\left(K_{0615}K_{0706}S_{0615}S_{0706} - K_{0616}K_{0710}S_{0616}S_{0710}\right) \\
&\quad + r_{38}\left(K_{0615}K_{0763b}S_{0615}S_{0763b} - K_{0616}K_{0763b}K_{1011}S_{0616}S_{0763b}S_{1011}\right) \\
&\quad - r_{37}\left(-K_{0615}S_{0615} + K_{0616}K_{1011}S_{0616}S_{1011}\right) \\
&\vdots
\end{aligned}
$$

S_i are the species amounts, K_i are the thermodynamic constants, r_j are the reaction rates. Species and reactions are indexed according to Stanford et al. (2013).

B.3.2. Julia Code

```
using BondGraphs, SBMLToolkit, ModelingToolkit, Catalyst, Graphs, Plots

# Source: 'Systematic Construction of Kinetic Models from Genome-Scale Metabolic Networks' (Stanford et al. 2013)
# https://doi.org/10.1371/journal.pone.0079195
sbmlfile = "yeast_model.xml"
sbmlurl = "https://doi.org/10.1371/journal.pone.0079195.s002"

# Download and process the SBML file
!isfile(sbmlfile) && download(sbmlurl, sbmlfile)
SBMLToolkit.checksupport_file(sbmlfile)
mdl = readSBML(sbmlfile, doc -> begin
    set_level_and_version(3, 2)(doc)
    convert_promotelocals_expandfuns(doc)
end)

# Remove reactions from reaction network that have a 'null' species
# i.e. remove 0 -> X and Y -> 0
isnullreaction(reaction) = isempty(reaction.substrates) || isempty(reaction.products)
function reducednetwork(network)
    newnetwork = make_empty_network(name=network.name)
    [addparam!(newnetwork, p) for p in parameters(network)]
    for rx in reactions(network)
        if  !isnullreaction(rx)
            [addspecies!(newnetwork, sub) for sub in rx.substrates]
            [addspecies!(newnetwork, prod) for prod in rx.products]
            addreaction!(newnetwork, rx)
        end
    end
    newnetwork
end

# Create the bond graph
rs = reducednetwork(ReactionSystem(mdl))
bg = BondGraph(rs)

# OUTPUTS
# Size of system
length(components(bg))  # 1019 components
length(junctions(bg))   # 1127 junctions
length(bonds(bg))       # 3813 bonds

# Adjacency matrix plot
A = adjacency_matrix(bg) # 2146×2146 SparseMatrix
plot(spy(A), size=(1000,800))
savefig("yeast_model_adjmat.png")

# Graph plot
plot(bg, size=(1000,800), names=[], title="")
savefig("yeast_model_bg.png")

# ODE equations
eqns = equations(bg)
length(eqns) # 293
eqns[3] # only displaying equation 3 for this example
```

B.3.3. Figures

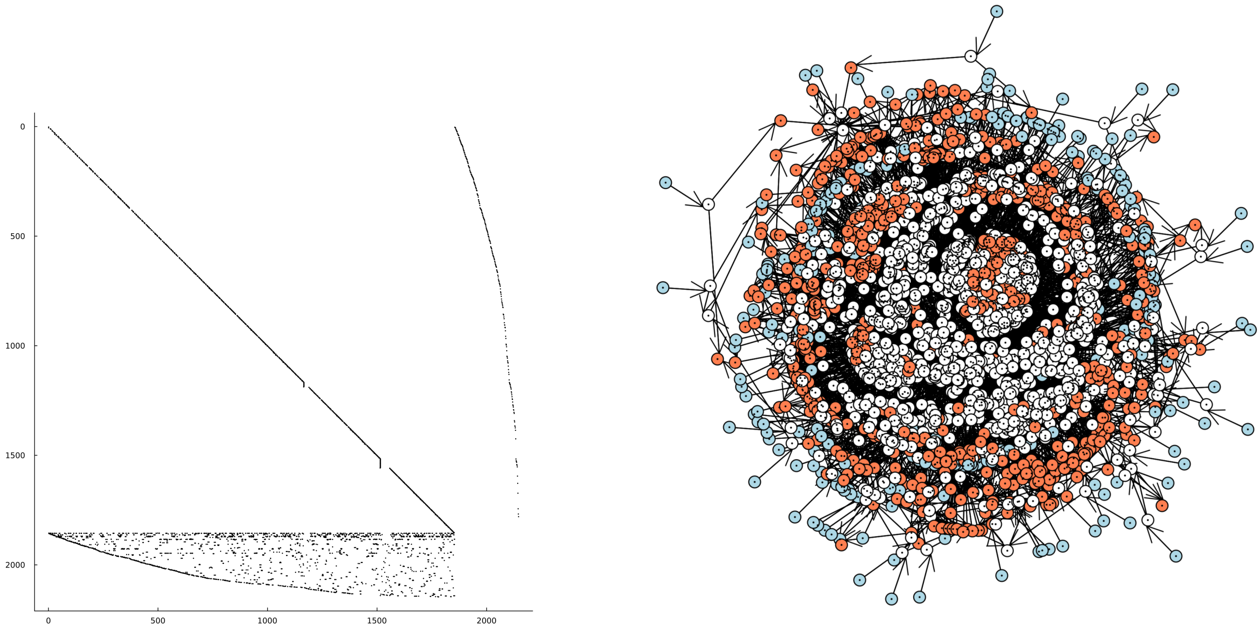

Fig. 1: Bond graph model of a yeast metabolic network (taken from Stanford et al., 2013). **Left:** 2146×2146 adjacency matrix for the yeast network bond graph. Axes correspond to the indices $a_{i,j}$ of the adjacency matrix, where $a_{i,j} = 1$ (black dot) means node i (component or junction) is connected to node j. **Right:** Graph plot of the yeast network bond graph. The graph contains 1019 components (blue for species, orange for reactions and transformers), 1127 junctions (white), and 3813 bonds.

References

Borutzky, W., editor (2011). *Bond Graph Modelling of Engineering Systems*. Springer New York, New York, NY.

Cudmore, P., Pan, M., Gawthrop, P. J., and Crampin, E. J. (2021). Analysing and simulating energy-based models in biology using BondGraphTools. *The European Physical Journal E*, 44(12):148.

Gawthrop, P. J. and Bevan, G. P. (2007). Bond-graph modeling. *IEEE Control Systems Magazine*, 27(2):24–45. Conference Name: IEEE Control Systems Magazine.

Gawthrop, P. J., Cursons, J., and Crampin, E. J. (2015). Hierarchical bond graph modelling of biochemical networks. *Proceedings of the Royal Society A: Mathematical, Physical and Engineering Sciences*, 471(2184):20150642. Publisher: Royal Society.

Gawthrop, P. J., Pan, M., and Crampin, E. J. (2021). Modular dynamic biomolecular modelling with bond graphs: the unification of stoichiometry, thermodynamics, kinetics and data. *Journal of The Royal Society Interface*, 18(181):20210478. Publisher: Royal Society.

Liebermeister, W. and Klipp, E. (2006). Bringing metabolic networks to life: convenience rate law and thermodynamic constraints. *Theoretical Biology and Medical Modelling*, 3(1):41.

Malik-Sheriff, R. S., Glont, M., Nguyen, T. V. N., Tiwari, K., Roberts, M. G., Xavier, A., Vu, M. T., Men, J., Maire, M., Kananathan, S., Fairbanks, E. L., Meyer, J. P., Arankalle, C., Varusai, T. M., Knight-Schrijver, V., Li, L., Dueñas-Roca, C., Dass, G., Keating, S. M., Park, Y. M., Buso, N., Rodriguez, N., Hucka, M., and Hermjakob, H. (2020). BioModels—15 years of sharing computational models in life science. *Nucleic Acids Research*, 48(D1):D407–D415.

Mason, J. C. and Covert, M. W. (2019). An energetic reformulation of kinetic rate laws enables scalable parameter estimation for biochemical networks. *Journal of Theoretical Biology*, 461:145–156.

Pan, M., Gawthrop, P. J., Cursons, J., and Crampin, E. J. (2021). Modular assembly of dynamic models in systems biology. *PLOS Computational Biology*, 17(10):e1009513. Publisher: Public Library of Science.

Pan, M., Gawthrop, P. J., Tran, K., Cursons, J., and Crampin, E. J. (2019). A thermodynamic framework for modelling membrane transporters. *Journal of Theoretical Biology*, 481:10–23.

Stanford, N. J., Lubitz, T., Smallbone, K., Klipp, E., Mendes, P., and Liebermeister, W. (2013). Systematic Construction of Kinetic Models from Genome-Scale Metabolic Networks. *PLOS ONE*, 8(11):e79195. Publisher: Public Library of Science.
